# Supplementary material for: In Vitro Cytotoxic Activity of Methanol Extracts of Selected Medicinal Plants Traditionally Used in Mexico against Human Hepatocellular Carcinoma
Source: Plants (Basel). 2022 Oct 27;11(21):2862. doi: 10.3390/plants11212862 (PMC9659118; doi:10.3390/plants11212862)
Supplement: Supplementary file 1 [file plants-11-02862-s001.zip › Informed-Consent-Form-for-Research.pdf]

INFORMED CONSENT FORM

This is an informed consent form related to a scientific research dealing with testing plant and endophyte extracts against tumor growth *in vitro*, using as a control of cytotoxicity human peripheral blood mononuclear cells from healthy adults. The sample volume will be 20 mL to 30 mL for each experiment (three experiments will be performed).

I, , confirm that (please check box as appropriate):

1.	I have read and understood the information about the project, as provided in the Information Sheet dated ________________.	☐
2.	I have been given the opportunity to ask questions about the project and my participation.	☐
3.	I voluntarily agree to participate in the research.	☐
4.	I understand I can withdraw at any time without giving reasons and that I will not be penalised for withdrawing nor will I be questioned on why I have withdrawn.	☐
5.	The procedures regarding confidentiality have been clearly explained (e.g. use of names, pseudonyms, anonymization of data, etc.) to me.	☐
6.	If applicable, separate terms of consent for interviews, audio, video or other forms of data collection have been explained and provided to me.	☐
7.	The use of the data in research, publications, sharing and archiving has been explained to me.	☐
8.	I understand that other researchers will have access to this data only if they agree to preserve the confidentiality of the data and if they agree to the terms I have specified in this form.	☐
9.	Select only **one** of the following:<br>• I would like my name used and understand what I have said or written as part of this study will be used in reports, publications and other research outputs so that anything I have contributed to this research can be recognised.	☐
	• I do not want my name used in this research.	☐
10.	I, along with the Researcher, agree to sign and date this informed consent form.	☐

________________________ ________________________

Participant's Signature Print Name

____

Date

________________________ ________________________

Researcher's Signature Print Name

____

Date

Form Created by AuthorizationForms.com
