# Supplementary material for: In Vitro Cytotoxic Activity of Methanol Extracts of Selected Medicinal Plants Traditionally Used in Mexico against Human Hepatocellular Carcinoma
Source: Plants (Basel). 2022 Oct 27;11(21):2862. doi: 10.3390/plants11212862 (PMC9659118; doi:10.3390/plants11212862)
Supplement: Supplementary file 1 [file plants-11-02862-s001.zip › Institutional Review Board approval JHEL.pdf]

UNIVERSIDAD AUTÓNOMA DE NUEVO LEÓN FACULTAD DE CIENCIAS BIOLÓGICAS

SUBJECT: Research project evaluation

Dr. Ricardo Gómez Flores:

By this means, you are informed that in a session held on April 26, 2022, the research project with registration number CI-09-2022 "Antitumor activity of methanolic extracts of Mexican plants and soil and endophytic microorganisms on tumor cell lines" was reviewed and approved.

The global scientific evaluation of this research project complies with technical-scientific quality, scientific relevance and impact, as well as with the criteria, guidelines and institutional requirements.

Sincerely yours,

Atentamente
"*ALERE FLAMMAM VERITATIS*"
Cd. Universitaria a 26 de abril del 2022

COMITÉ DE INVESTIGACIÓN

Dr. Juan Francisco Contreras
Presidente

Dra. María Julissa Ek Ramos
Secretario

Pedro de Alba y Manuel L. Barragán s/n, Cd. Universitaria, San Nicolás de los Garza, Nuevo León, México.
Tel. (81) 83294110 ext. 6432
